# Role of B Cell Development Marker CD10 in Cancer Progression and Prognosis

**DOI:** 10.1155/2016/4328697

**Published:** 2016-11-14

**Authors:** Deepshikha Mishra, Sunita Singh, Gopeshwar Narayan

**Affiliations:** ^1^Department of Molecular and Human Genetics, Banaras Hindu University, Varanasi 221 005, India; ^2^Department of Zoology, Mahila Mahavidyalaya, Banaras Hindu University, Varanasi 221 005, India

## Abstract

The human CD10 antigen is a single pass, type II transmembrane, 100 kD cell surface glycoprotein belonging to peptidase M13 family. Identified in common acute lymphoblastic leukemia as a cancer specific antigen, CD10 is a cell surface ectoenzyme widely expressed on different types of cells. Earlier, it was used only as a cell surface marker to identify and differentiate between haematological malignancies. Later, reported to be present in various malignancies, it is thought to play significant role in cancer development and progression. Regulated expression of CD10 is necessary for angiogenesis and so forth. However its expression level is found to be deregulated in different cancers. In some cancers, it acts as tumor suppressor and inhibits tumor progression whereas in others it has tumor promoting tendency. However, its role in tumorigenesis remains unclear. This review summarises structural features, functions, and probable role of CD10 in cancer development.

## 1. Introduction

Cluster of differentiation CD10, neprilysin, common acute lymphoblastic leukemia antigen (CALLA), neutral endopeptidase (NEP), enkephalinase, or EC 3.4.24.11 is a 90–110 kDa cell surface type II integral membrane protein of M13 family [1]. NEP protein comprises of three domains: a short cytoplasmic N terminal domain, a transmembrane hydrophobic domain, and a large extracellular domain having catalytic activity [2]. Phylogenetic analysis of zinc-metalloenzymes of M13 family shows two major subsites: a comparatively invariant S1′ subsite responsible for their strong preference for hydrophobic residues and variation rich S2′ subsite, a key determining factor for substrate specificity of M13 peptidases [3].

**Figure 1:**
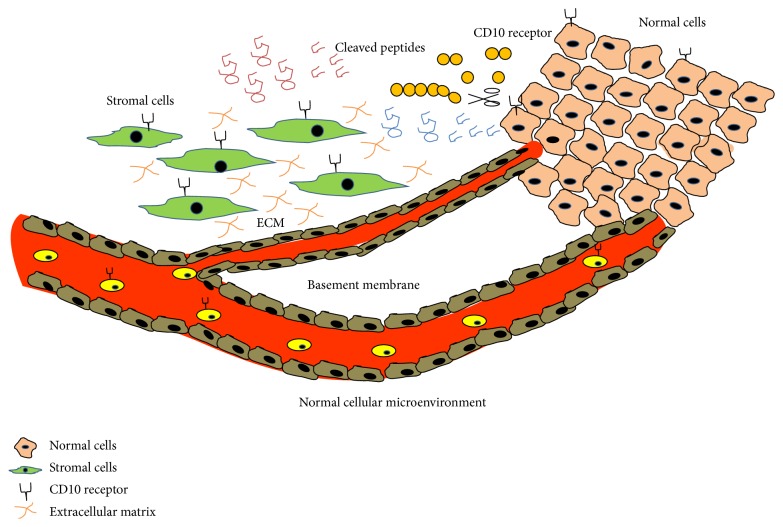
Role of neutral endopeptidase CD10 in inactivating multiple physiologically active peptides like endothelin-1, bombesin, bradykinin, Leu- and Met-enkephalins, atrial natriuretic factor, oxytocin, neurotensin, and bombesin-like peptides present in the normal cellular microenvironment.

Common acute lymphoblastic leukemia antigen (CALLA) is expressed specifically in early lymphoid progenitor stages showing immature phenotype that suggests its role in lymphoid cell development and differentiation. It was originally identified to be present on acute lymphoblastic leukemic cells [4, 5] and hence was called common acute lymphoblastic leukemia antigen, albeit was later found to be expressed in a variety of cells, including prostate, kidney, intestine, endometrium, adrenal glands, and lung. Its presence on other cells suggests its varied biological role not restricted specifically to haematological malignancies. It acts by cleaving amino side peptide bonds of the hydrophobic amino acids causing inactivation of a range of physiologically active neuropeptides. CD10 metabolises biologically active peptides like bradykinin, oxytocin, atrial natriuretic factor, substance P, bombesin, endothelin-1, Leu- and Met-enkephalins, neurotensin, and so forth, resulting in reduced concentrations of functionally active neuropeptide for receptor ligand signalling, neurotransmitter levels modulation, blood pressure control, reproduction, and so forth [6, 7].  Figure 1 shows the role of CD10 in inactivating a broad range of physiologically active peptides.

Studies have shown the role of CD10 in Alzheimer's disease, ageing, cardiac disorders, and some other diseases; however, there is a scarcity of literature to establish a clear relation between CD10 and cancer development. Various techniques like immunohistochemistry, fine needle aspiration cytology (FNAC) staining, and enzyme-linked immunosorbent assay are used for its detection. The differential expression of CD10 appears to be very interesting feature from a diagnostic point of view which can be exploited further as a marker for good and bad prognosis between different stages of cancer.

This review is aimed at providing therapeutic and diagnostic insights and further evaluates CD10 contribution in cancer progression. In addition, this review also summarises CD10 expression status in different tumors and its biological significance. Herein, we have summarised CD10 involvement and role in cancer progression. We have studied the expression of CD10 in different tumors to determine whether it could serve as a progression marker for tumor and its prognosis. Finally, we have reviewed its clinical correlation with cancer progression and relevance as a cancer biomarker.

## 2. Molecular and Structural Features of CD10 (NEP)

Present on chromosome 3 at 3q25.2 cytogenetic band, CD10 (NEP) is present as a single copy of more than 45 kb. CD10 shows alternative splicing in the 5′ untranslated region, producing four different mRNA transcripts though the coding region remains unaffected. Biologically, its main function is to metabolise polypeptides of up to 30 amino acids preferentially by cleaving peptides between hydrophobic residues. Three-dimensional secondary structural model of CD10-enzyme shows presence of 400 residues' active site located in a central pocket [8] having a glutamate-active (E646) site responsible for catalysis and two histidine-active (^583^HExxH^687^) sites, responsible for cofactor zinc binding [9]. It shows a highly conserved sequence homology with other species with only minor changes in amino acid sequences. CD10 activity is inhibited by phosphoramidon, which binds to its active enzymatic site. Phosphoramidon is a metabolite isolated from* Streptomyces*, initially identified as a thermolysin inhibitor [8]. CD10, present on pre-B cell, is transiently expressed during different stages in B cell maturation stages and it disappears in mature B cells. This suggests that CD10 might play a role in pre-B cell maturation and differentiation [10]. Apart from pre-B cells, it is expressed by endometrial stromal cells [11], liver [12], and stomach and colon [13] cells.

## 3. CD10 Expression in Normal B Cell Development

Time of appearance and disappearance of cell surface markers can be very helpful in tracing the events involved in the course of a cell development. The mature and immature B cells can be easily identified at the developmental stage by the presence or absence of immunoglobulin, which is not (or is poorly) present on the cell surface of the immature B cells but is expressed later on the differentiated later stages. A battery of other clusters of cell surface differentiation markers like CD10, CD19, CD20, CD21, CD24, CD34, and CD38 help in easy identification of stages in B cell development [14]. Out of numerous CD markers, CD10 is of prime importance as it is a common lymphoid progenitor (CLP) of early stages of differentiation of B (E-B) stage [15]. An increase in the expression of CD10 cell surface marker is found to be directly proportional to the differentiation potential of B cells and further represents developmental commitment and progression [16]. Expression of CD10 on cell surface has prime clinical importance as it can be easily quantified through flow cytometry. A deregulated expression of CD10 also plays a role in infant leukemias of early B developmental stages [17] and can effectively serve as target for clinical intervention and targeted drug designing. CD10 expression on immature B cell surface is critical for its development; however, now it is found to be expressed by a variety of other cell types. CD10 was found to react with majority of non-T-cell ALL patients and not with normal hematopoietic cells [18]; hence it is widely used for distinguishing most cases of ALL from other hematologic malignancies. CD10 is commonly used in the flow cytometric diagnosis and monitoring of haematological malignancies of B cell origin, mature and blastic stage categorisation, and further MRD detection [19–21].

## 4. CD10 Expression in Cancers

The role of neprilysin, also known as CD10, has been correlated with many cancers; however, their exact roles in tumor progression and resistance are not well established. However majority of malignancies show an upregulated expression of CD10 and its correlation with higher tumor stage and severity; hence it can be concluded that CD10 behave as double edge swords. Stromal cells CD10 expression could be one of the reasons behind observed behaviour.

CD10 expression is considered an adverse prognostic factor in lung adenocarcinoma patients where hypoxic condition present in the microenvironment is considered one of the key reasons behind CD10 upregulated expression in the tumor stroma [22]. Interestingly, NF-*κ*B expression shows inverse correlation with CD10 expression and is considered an adverse prognostic factor for relapse after radical prostatectomy in prostate cancer [23]. Expression of CD10 surface antigen is correlated with therapeutic resistance by being refractory to drugs and radiation in head and neck squamous cell carcinoma (HNSCC) [24]. A higher expression in the invasive front in colorectal cancer tumor samples shows CD10 involvement in cancer development and progression [25]. The expression of CD10 in seminomas, intratubular germ cell tumor and in precursors of germ cell tumors and loss after differentiation can be used as important marker to differentiate seminoma and testicular tumor [26]. Increased stromal CD10 expression is significantly related with an increasing tumor grade in breast cancer; further its expression is also found to be higher in unfavourable group [27]. An increased level of expression was found in patients with liver metastasis and advanced cancer stages [28]. A new study shows upregulation of CD10 expression by Twist1 and its direct correlation with cell migration and anchorage-independent tumor growth in esophageal squamous cell carcinoma cells [29]. It is also widely expressed in well-differentiated to moderate to poorly differentiated samples in hepatocellular carcinoma and the canalicular staining can be exploited as a highly specific hepatocytic differentiation marker [30, 31]. It shows specifically strong expression in basal cell carcinoma (BCC) with null expression in deeply infiltrative BCCs making it a diagnostically important marker [32]. A more aggressive phenotype expression with higher malignant potential is associated with CD10 expression in prostate cancer [33]. Pancreatic tumors showed differential expression of NEP/CD10 which was involved in tumor cell proliferation activity of pancreatic cancer cells [34]. Stromal CD10 expression was strongly correlated with higher tumor grade and estrogen receptor negativity; however no correlation was found with progesterone receptors, Her2 status, lymph node, tumor size, histological subtype, and so forth. Further, stromal cell CD10 expression was also found to be associated with decreased survival [35]. Expression of CD10 showed significant correlation with high proliferative index, tumor size, and metastasis; further a membrane expression correlated with poor differentiation [36]. In colorectal carcinoma, CD10 expression is correlated with liver metastasis and can be used as good predictor [37]. However in urothelial tumors, cytoplasmic staining was predominant with moderate to strong expression in majority of neoplasms [38]. CD10 overexpression has also been associated with colorectal cancer development and progression [39]. In gastric cancer, stromal cells CD10 expression correlated with hallmark feature of cancer like invasion and metastasis [40]. A specific pattern of increasing stromal CD10 expression in benign lesions and malignant phyllodes tumors suggests its usefulness in assessing tumor [41]. In invasive breast cancer, stromal expression of CD10 highlights high grade, estrogen receptor negativity, and poor prognosis [42] and can be used as a predictor of disease outcome [43]. In melanomas CD10 protein expression level detected by immunohistochemistry was found to be more in advanced primary tumors and metastatic melanomas than primary tumors [44]. A differential expression pattern with an upregulated level of CD10 was found during the process of metastasis in melanomas of skin and can be diagnostically used to differentiate between primary and metastatic melanomas [45]. CD10 protein expression level was found to be decreased in poorly differentiated type of human adenocarcinoma by western blot analysis as compared to normal epithelial cells of stomach and colon [13]. On the contrary, its expression is not detected in normal thyroid tissue but is found to be highly expressed in follicular variants of papillary thyroid cancer (PTC) [46] and advanced stages of PTC [47]. In the process of prostate cancer development, a decreased or total loss of CD10 expression is an early and frequent event and differentiates hormone sensitive and refractory cases [48]. This CD10 loss is known to play critical role in the development of androgen-independent prostate cancer by using mitogenic neuropeptides as an alternative source for cell proliferation in the place of androgen [49]. A high level of CD10 expression in primary tumor sample of prostate cancer is significantly associated with larger size of metastases, early death [50], and aggressive phenotype with a higher malignancy rate [33] and can be effectively used for stratifying prostate cancer outcome. A decreased expression of CD10 is correlated with higher proliferation and invasion in breast cancer [51].  Table 1 shows the correlation between CD10 expression level and its prognostic implications as reported in different cancers.

**Figure 2:**
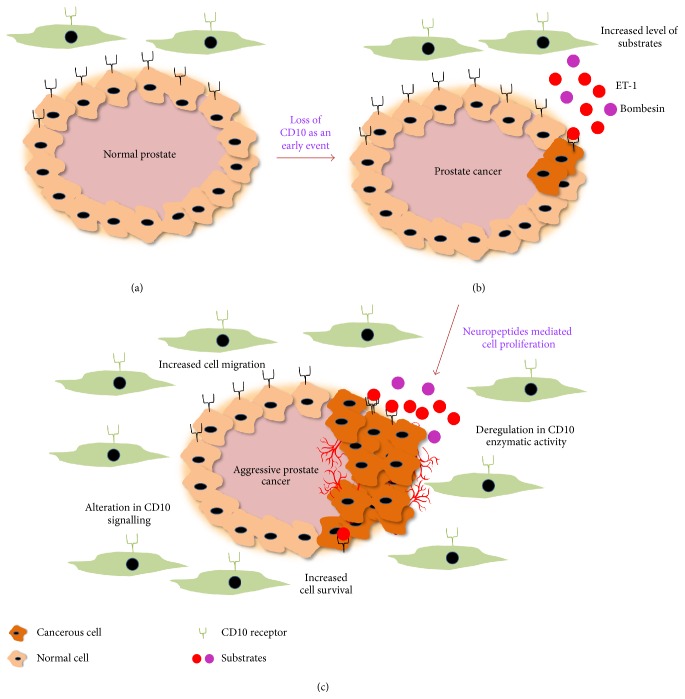
Prostate cancer model showing (a) normal prostate, (b) early prostate cancer, and (c) late stage aggressive prostate cancer. High amount of peptides accumulated in the cellular microenvironment facilitates neoplastic transformation and further progression. Excess neuropeptides like bombesin and endothelin-1 (ET-1) as substrate deregulate multiple signalling pathways and make the cancer aggressive and treatment refractory.

Figure 2 shows the generalised model for cancer development using prostate cancer as an example. Total loss or decreases in CD10 expression promote peptide-mediated aberrant cell proliferation by high amount of accumulated peptide concentrations.

## 5. Probable Mechanism and Pathways

The CD10, also known as neprilysin, primarily with its enzymatic activity acts on multiple downstream target; this might contribute to the development of diseased state. One of the most plausible explanations behind CD10 involvement in tumorigenesis is by the enhanced accumulation of peptides that are cleaved by CD10, which leads to the proliferation of undifferentiated cells. Secondly, CD10 may also act via altering signalling pathways associated. While it targets outside the cell via its enzymatic activity, it targets at intracellular level by interacting with various signalling pathways. It is known from the available literature that CD10 inactivates a diverse range of physiologically active neuropeptides by cleaving amino terminal peptide bonds of hydrophobic amino acids [1]. Thereby it clears physiologically active neuropeptides present in the microenvironment available for cell signalling. So it is expected that a decreased CD10 expression might be responsible for tumor progression by presence of higher peptide concentrations available for higher cell signalling in tumor milieu which will further facilitate tumor proliferation. CD10 is known to behave as a tumor suppressor by inhibiting various events contributing to neoplastic progression. A few studies on prostate cancer show CD10 role as tumor suppressor and involvement in cancer progression and the development; however the exact mechanism underlying is yet to be explored. A decreased or loss of CD10 expression is a frequent and early event in prostate cancer development [49]. It inhibits cell migration via protein-protein interaction with tyrosine phosphorylated Lyn kinase forming neprilysin-Lyn-phosphatidylinositol-3-kinase protein complex. This complex blocks focal adhesion kinase and phosphatidylinositol-3-kinase interaction competitively, inhibiting cell migration [54]. It also inhibits tumorigenesis in prostate cancer via reducing FGF-2-mediated angiogenesis; it negatively regulates angiogenesis by proteolytically inactivating fibroblast growth factor-2 [55, 56]. It also interacts with endogenous PTEN tumor suppressor, recruiting it to cell membrane which causes prolonged PTEN protein stability and phosphatase activity. This increased activity results in constitutive downregulation of AKT [57]. CD10-positive subpopulation in head and neck squamous cell carcinoma acquires cancer stem cell (CSC) property and expresses higher level of CSC marker OCT3/4. An elevated level of CD10 and OCT3/4 results in increased tendency to form tumors and spheres implicated in therapeutic resistance and refractory HNSCC [24]. MUC2, a glycoprotein, forms insoluble protective mucous barrier inside the gut lumen but its reduced expression along with CD10 overexpression is reported to play a role in development of and progression of colorectal cancer [39]. In cervical [52] and ovarian carcinoma [53], a decreased CD10 expression promotes cancer progression. A loss of CD10 expression by DNA methylation of promoter is one of the factors causing lymphoid malignancies [58]; a similar mechanism can play a crucial role as an early event in other malignancies also. Some of the important signalling pathways involved in CD10 mediated cancer progression are summarised in Table 2.

## 6. CD10: Chemotherapy and Diagnostic Implications

Chemotherapy is known to modulate CD10 expression in some cancers. In NALM-1 pre-B leukemic cell line, doxorubicin and PMA downregulated CD10 expression [59] whereas prednisone significantly reduced CD10 and CD34 expression [60]. Breast cancer stromal CD10 expression profile changes with neoadjuvant anthracycline-based chemotherapy [61]. Further an expression level after chemotherapy correlates to poor clinical response and a decreased level shows complete or partial clinical response [27]; also stromal CD10 expression significantly correlated with increasing tumor grade, mitotic rate, ER negativity, Her2neu positivity, and worse prognosis which suggests its role as a routine prechemotherapy marker in breast carcinoma [35]. In ovarian cancer, CD10 overexpression increased paclitaxel susceptibility and reduced tumorigenesis in vivo also [53]. In head and neck squamous cell carcinoma (HNSCC), CD10-positive cell population was found to be more refractory to radiation and chemotherapeutic drugs like cisplatin and fluorouracil than the CD10-negative population. Also, CD10-positive population possessed cancer stem cell features and expressed a higher level of cancer stem cell markers OCT3/4 with enhanced tendency to form spheres in both in vitro and in vivo tumors, which suggests a cancer stem cell like proliferative property in these cancer cells [24]. Chemotherapeutic drug doxorubicin is known to decrease the expression of CD10 [59]; one of the most plausible reasons behind it could be presence of significant binding sites between doxorubicin and CD10 as shown by molecular docking analysis [62].

CD10 qualifies to be of high diagnostic utility by being able to discriminate between hepatocellular carcinoma (HCC) and metastatic carcinoma of the liver [30]. CD10 expression is used as marker to diagnose follicular carcinoma with follicular variant of papillary thyroid carcinoma [46]. However, it cannot be used to differentiate between benign and malignant cases but shows strong positivity in papillary carcinoma [63]. It can also be used as an additional marker for the diagnosis of renal malignancies [64]. Its role is also implicated in differential diagnosis of renal cancer, always negative gynecologic clear cell carcinoma types and metastatic clear cell carcinoma types [65]. CD10 marker is of high importance in endometrial stromal neoplasms and can be used in a panel with desmin and alpha-inhibin [66]. Interestingly, endometrial mixed carcinoma cells expressing CD10 show long survival [67]. It is differentially expressed in small cell carcinomas of the lung (SCLC), being minimally expressed in bronchoalveolar and large cell carcinoma cell lines, whereas it is highly expressed in squamous, adenosquamous, and adenocarcinoma cell lines [68]. In totality, CD10 serves as a reliable and sensitive marker of normal and cancerous tissues.

## 7. Conclusion and Perspective

CD10 presence on epithelial cells as well as on surrounding stromal cells in tumor milieu might be a driving force in cancer progression. Enzymatically cleaved peptides can act via autocrine or paracrine signalling favouring molecular dysregulation in the tumor microenvironment. A deregulated CD10 expression by stromal cells and tumor cell may lead to cancer growth and progression disturbing the cell normal microenvironment. Its remarkable presence on different cancerous cells might suggest its role as a good progression marker and can be used widely for differentiating between treatment favourable and adverse stages and can therefore lead to an important role in the category of tumor progression marker.

The classical role of this cell surface enzyme is to hydrolyze different peptides involved in varied biological processes like tissue remodelling, embryogenesis, angiogenesis, and so forth, present in the extracellular matrix (ECM). A regulated CD10 expression by epithelial and stromal cells is necessary for proper homeostasis maintenance in the matrix and deregulation of this balance results in cancer and Alzheimer's disease by accumulating in the cell microenvironment which results in generation of key factors contributing or inhibiting deregulated cell proliferation, angiogenesis, and migration. Besides their role as peptide degraders, CD10 interact elegantly with the intracellular signalling pathways like PI3-K pathway, Fak-Src pathway, and so forth, and this communication activates downstream molecules.

Our knowledge about diverse roles of CD10 in the cancer microenvironment is expanding after improved understanding of the CD10 signalling behind cancer progression and resistance. However, the differential expression of CD10 in cancer and varied outcomes remain a problem. Future studies on meta-analysis of CD10 expression will be extremely helpful.

In conclusion, CD10 can be a very useful progression marker and an attractive molecular target for targeted therapy designing. Its routine expression analysis along with other markers might be very helpful in cancer diagnosis and treatment response. Even though its expression in some cases is related with better treatment response, CD10 expression is biased towards cancer proliferation and progression. This behaviour suggests that CD10 behaves as dual edge sword and depending upon the peptides present in tumor microenvironment modulates cancer progression accordingly. This review explores the role of CD10 in cancer detection and prognosis and its utility as an important marker for a better detection when used with other progression markers. However, its dual nature warrants more detailed and extensive investigations in this field.

## Figures and Tables

**Table 1:** CD10 expression status in different cancers.

Cancer	CD10 level	Prognosis	References
Prostate cancer	Decreased/total loss of CD10 expression	Androgen-independent progression	[48, 49]
Prostate cancer	High level of CD10	Aggressive phenotype, higher malignancy rate, larger metastases, early death	[33, 50]
Breast cancer	Stromal CD10 expression	Poor prognosis, estrogen receptor negativity, high grade	[35, 43]
Melanomas	High CD10 expression	Advanced stage, higher metastasis	[44, 45]
Follicular papillary thyroid cancer	High CD10 expression	Bad	[46]
Papillary thyroid cancer	High CD10 expression	Advanced stage	[47]
Adenocarcinoma of stomach and colon	Decreased CD10 expression	Poor differentiation	[13]
Head and neck squamous cell carcinoma	Increased CD10 expression	Therapeutic resistance	[44]
Colorectal cancer	Increased CD10 expression	Higher invasion	[25]
Colorectal cancer	Serum CD10 expression	Liver metastasis	[28]
Esophageal squamous cell carcinoma	Upregulated CD10 expression	Poor disease-free survival and overall survival	[29]
Pancreatic endocrine tumors	Membranous expression of CD10	Poor differentiation, high proliferative index, low microvascular density, large tumor size, metastasis, poor survival	[36]
Cervical carcinoma	Decreased CD10 expression	Higher proliferation and invasion	[52]
Ovarian carcinoma	Increased CD10 expression	Suppressing progressive potential	[53]
Gastric carcinoma	Stromal cells CD10 expression	Differentiated carcinoma, high depth of invasion, lymph node metastasis	[40]

**Table 2:** Main signalling pathways involved in CD10 mediated malignancies.

Cancer	Signalling pathway	Reference
Prostate cancer	CD10-FAK kinase interaction	[54]
	FGF-2-mediated angiogenesis	[55, 56]
Non-small cell lung cancer	Hypoxia induced stromal CD10 upregulation	[22]
Head and neck squamous cell carcinoma	Increased expression of OCT3/4 by CD10-positive population	[24]
Esophageal squamous cell carcinoma	Twist1 mediated CD10 upregulation	[29]
Prostate cancer	Decreased CD10 expression with high NF-*κ*B expression.	[23]
Prostate cancer	CD10 loss mediated Akt activation	[57]
Colorectal cancer	CD10 overexpression with MUC2 reduced expression	[39]
Leukemias	DNA methylation of promoter region of CD10	[58]
